# Relationship between Knockdown Resistance, Metabolic Detoxification and Organismal Resistance to Pyrethroids in *Anopheles sinensis*

**DOI:** 10.1371/journal.pone.0055475

**Published:** 2013-02-06

**Authors:** Daibin Zhong, Xuelian Chang, Guofa Zhou, Zhengbo He, Fengyang Fu, Zhentian Yan, Guoding Zhu, Tielong Xu, Mariangela Bonizzoni, Mei-Hui Wang, Liwang Cui, Bin Zheng, Bin Chen, Guiyun Yan

**Affiliations:** 1 Program in Public Health, College of Health Sciences, University of California Irvine, Irvine, California, United States of America; 2 Department of Pathogen Biology, Nanjing Medical University, Nanjing, China; 3 Department of Pathogen Biology, Bengbu Medical College, Anhui, China; 4 Institute of Entomology and Molecular Biology, College of Life Sciences, Chongqing Normal University, Chongqing, People's Republic of China; 5 Division of Malaria Control, Jiangsu Institute of Parasitic Diseases, Wuxi, China; 6 National Institute of Parasitic Diseases, Chinese Center for Disease Control and Prevention, Shanghai, People's Republic of China; 7 Department of Entomology, the Pennsylvania State University, University Park, Pennsylvania, United States of America; Swedish University of Agricultural Sciences, Sweden

## Abstract

*Anopheles sinensis* is the most important vector of malaria in Southeast Asia, including China. Currently, the most effective measure to prevent malaria transmission relies on vector control through the use of insecticides, primarily pyrethroids. Extensive use of insecticides poses strong selection pressure on mosquito populations for resistance. Resistance to insecticides can arise due to mutations in the insecticide target site (target site resistance), which in the case of pyrethroids is the *para*-type sodium channel gene, and/or the catabolism of the insecticide by detoxification enzymes before it reaches its target (metabolic detoxification resistance). In this study, we examined deltamethrin resistance in *An. sinensis* from China and investigated the relative importance of target site *versus* metabolic detoxification mechanisms in resistance. A high frequency (>85%) of nonsynonymous mutations in the *para* gene was found in populations from central China, but not in populations from southern China. Metabolic detoxification as measured by the activity of monooxygenases and glutathione S-transferases (GSTs) was detected in populations from both central and southern China. Monooxygenase activity levels were significantly higher in the resistant than the susceptible mosquitoes, independently of their geographic origin. Stepwise multiple regression analyses in mosquito populations from central China found that both knockdown resistance (*kdr*) mutations and monooxygenase activity were significantly associated with deltamethrin resistance, with monooxygenase activity playing a stronger role. These results demonstrate the importance of metabolic detoxification in pyrethroid resistance in *An. sinensis*, and suggest that different mechanisms of resistance could evolve in geographically different populations.

## Introduction

The mosquito *Anopheles sinensis* is the major malaria vector in China and other Southeast Asian countries [1]–[6]. The World Health Organization (WHO) defines vector control as one of the four basic and most effective measures to prevent malaria transmission. Vector control relies primarily on the use of insecticides through insecticide-impregnated bednets and indoor residual spray [7]. Chemical insecticides have been extensively used since the 1940s to control mosquito vectors, and historically, four major categories of insecticides have been sequentially applied: organochlorines, organophosphates, carbamates and pyrethroids [8]–[10]. In the past decade, pyrethroids have become the preferred choice among the currently WHO approved compounds because of their low toxicity to humans, high efficacy against mosquito vectors and short residual action [11].

Synthetic pyrethroids, particularly permethrin and deltamethrin, are widely used for malaria vector control worldwide [12], [13], where the use of impregnated bednets has been shown to be very effective in reducing malaria transmission [14], [15]. Pyrethroids are also used for agricultural pest control. In China, extensive use of insecticides for agricultural pest and public health disease vector control has posed intensive selection pressure on mosquitoes [12], [16]. High levels of resistance to deltamethrin have been reported in *An. sinensis* populations from China and Korea [12], [17].

It is crucial to understand the mechanism of insecticide resistance in order to establish reliable resistance diagnosis methods and aid in resistance management. Pyrethroid resistance in malaria vectors has been mostly studied in the major African malaria vector, *An. gambiae* [18]–[23]. Two major resistance mechanisms are known: a) mutations in the *para*-type sodium channel gene, the target site of pyrethroids, causing a change in affinity between the insecticide and its binding site that reduces sensitivity to the insecticide; and b) metabolic detoxification of pyrethroids before they reach their target site by detoxification enzymes (the multigene family of P450 monooxygenases, esterases, and glutathione-S-transferases) [24].

Several point mutations in the *para*-type sodium channel gene have been identified in *Anopheles* mosquitoes in association with pyrethroid resistance. The most common mutation conferring knockdown resistance (*kdr*) is a mutation at position 1014 causing a change from Leucine to either Phenylalanine (L1014F) or Serine (L1014S) [17], [25]–[31]. In *Musca domestica* [32] and *Haematobia irritans* [33] a mutation at position 918 (M918T) of the *para* gene was identified as being correlated with very high levels of pyrethroid resistance (super *kdr* phenotype) [32], [34]. Metabolic detoxification has been linked to elevated levels of P450 monooxygenases, esterases, and glutathione-S-transferases (GSTs) [24], [35]–[37].

In the present study we assessed the level of pyrethroid resistance in *An. sinensis* from five localities in southern and central China and examined the frequency of *kdr* mutations and the levels of detoxification enzymes in the phenotyped mosquitoes.

## Materials and Methods

### Study Sites

**Figure 1:**
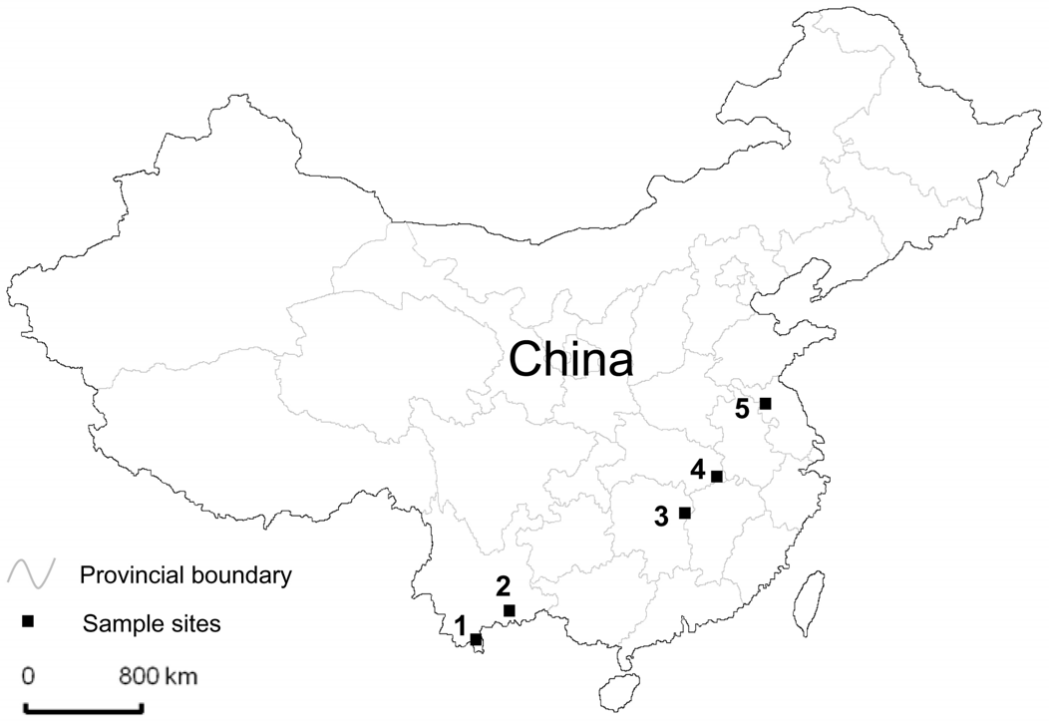
A map of China showing the distribution of mosquito sampling sites. Site name: 1, Mengla county (Yunnan Province); 2, Yuanyang county (Yunnan Province); 3, Liuyang county (Hunan Province); 4, Wuxue county (Hubei Province); and 5, Sihong city (Jiangsu Province).

*Anopheles sinensis* larvae and pupae were collected from five different geographical sites in China, including Yunnan (Mengla and Yuanyang), Hunan (Liuyang), Hubei (Wuxue) and Jiangsu (Sihong) provinces (Fig. 1). The Yunnan and Hubei sites are malaria endemic. The Hunan site was historically endemic. Jiangsu has been experiencing sporadic *P. vivax* malaria outbreaks. Since the 1980s in China, deltamethrin impregnated nets (ITNs) and indoor residual spraying (IRS) have been widely used against mosquitoes, as well as other insects [38]. Rice is the major agricultural crop in these study sites. There are two or three rice harvests each year in the Yunnan sites, but only one harvest per year in the Hunan, Hubei and Jiangsu sites. Due to severe insect pest damage to the rice crop, insecticide use for rice pest control has been very intensive, with several rounds of sprays in each growing season. Pyrethroids are commonly used for agricultural pest control in study areas, but other insecticides such as organic phosphates and carbamates are also being used [16].

### Mosquito Sampling

In each site, we collected about 1,000 larvae and pupae from more than 100 larval habitats, mostly rice fields and small ponds with aquatic plants, using the standard 350 ml dippers. Mosquito larvae and pupae were reared to adults for bioassay in order to eliminate the influence of mosquito age and blood feeding history on resistance. After identifying mosquitoes to species morphologically, larvae and pupae of *An. sinensis* were kept in plastic storage bins and transported to a local rearing shelter close to the collection site. Larvae and pupae were reared to adults under local environmental conditions. The emerged female adults were used in deltamethrin resistance bioassay [39], enzyme activity assay and *kdr* allele genotyping assays. Mosquito sampling and bioassays were conducted in July–August 2010 for the two sites in Yunnan province. Collections from the Hunan, Hubei and Jiangsu study sites were conducted in July–August 2011. A laboratory susceptible strain that has been maintained in the insectary of the Jiangsu Institute of Parasitic Diseases in Wuxi, China, for more than 10 years with no insecticide exposure was used as the reference susceptible strain.

### Insecticide Susceptibility Bioassay

Female adult mosquitoes directly reared from field-collected larvae and pupae, 3 days post adult emergence, were individually tested for susceptibility to deltamethrin, using the standard WHO tube bioassay with 0.05% deltamethrin test papers [39]. For each population, about 100–150 female mosquitoes were tested. Paraffin oil-treated papers without insecticide were used as control. The knockdown time of individual mosquitoes was recorded as minutes within one hour. After 1-hr exposure to the diagnostic concentration of deltamethrin (0.05%), mosquitoes were transferred to recovery cups and maintained on 6% sucrose solution for 24 hrs. Mosquito survival status was examined every 3 hrs, and dead mosquitoes were immediately collected and preserved at −20°C prior to metabolic enzyme analysis. Mosquitoes were considered dead if they were motionless, even when they were mechanically stimulated, following the method of González Audino [40]. This is reasonable because motionless mosquitoes after bioassay exposure rarely recovered and survived. One leg of each mosquito was also removed and preserved individually in 95% alcohol for subsequent DNA analysis. The intact tissue of the remaining mosquito body was immediately tested for metabolic enzyme activity [41], [42].

### Metabolic Enzyme Activity Assays

Individual females were homogenized in a 1.5-ml tube with 200 µl of phosphate KPO_4_ buffer (0.25 M, pH 7.2) and then diluted by adding 1,300 µl of phosphate buffer. The tube was mixed and centrifuged, and the supernatant was used to test the activity of GSTs and monooxygenases [25], [43], [44]. GST and monooxygenase assays were carried out in duplicate. A total of 300 µl reduced glutathione solution and 300 µl cDNB solution was added to 300 µl of mosquito supernatant. The absorbance was measured immediately, and then every minute for 5 minutes at 340 nm, using 0.25 M KPO_4_ buffer as the negative control. The change in absorbance was converted to enzyme activity using the CDNB extinction coefficient of 0.0096 uM^−1^cm^−1^ for this reaction, and the estimated protein content of the supernatant was measured by the Bradford method [45]. For the monooxygenase activity assay, a total of 8 µl of the 7-ethoxycoumarine (7-EC) solution was added to 400 µl of mosquito supernatant and samples were incubated at 30°C for 4 hours. The reaction was stopped by the addition of 560 µl of glycine buffer (pH 10.4, 0.1 mM) and sample absorbance was measured at 450 nm using 0.25 M KPO_4_ buffer as a negative control. The OD values were converted into concentration by using a standard regression based on a serial dilution of 7-hydroxycoumarin and its relevant OD values. The value of monooxygenase content was calculated for each mosquito.

### Mosquito DNA Extraction

One leg of each mosquito was used for DNA extraction with the Fast Tissue-to-PCR Kit (Fermentas). Briefly, each mosquito leg was placed at the bottom of a 500 µl Eppendorf tube. A total of 50 µl of tissue lyses solution and 5 µl of protein K solution were added and incubated at 55°C for 20 min, followed by 10 minutes at 95°C. After the incubations, 50 µl of neutralization solution was added and mixed by vortexing. The neutralized tissue was then centrifuged at 14,000 rpm for 10 min. Extracted DNA was stored at 4°C or used immediately for PCR.

### Molecular Identification and Detection of *kdr* Mutation

Molecular identifications of *An. sinensis* species were conducted by using species-specific primers and amplification of the ITS2 and 28S rDNA regions (D1 and D2) [46]. To determine point mutations of the *para*-type sodium gene at positions 1014 and 918, we first amplified a 325 bp fragment of the *para*-type sodium gene including position 1014, and then a 450 bp fragment including position 918 (Table 1). PCR primers were designed based on the *An. sinensis* sequences of the DIIS6 region of the *para*-type sodium gene (Genbank acc. no. DQ334052). PCR products were directly sequenced in both forward and reverse directions using the same primers. Direct sequencing was performed by Genewiz Inc (South Plainfield, NJ) on 150 field-derived mosquitoes. Based on data obtained from these individuals, allele specific amplification (AS-PCR) primers were designed to detect four mutations (TTG, TTT, TGT and TTC). To increase allele specific amplification, each primer was designed to incorporate a single base mismatch at third base position from the 3' end terminus. The DNA sequences were used as a gold standard to determine the sensitivity and specificity of the AS-PCR method.

**Table 1:** Primer sequence of multiplex allele-specific PCR for the detection of mutations in codon 1014 of *para*-type sodium channel gene and super *kdr* mutation in codon 918 in *Anopheles sinensis.*

Name	Primer sequence (5' −> 3')	bp
TTG-F	CTGTGGTAATTGGAACCTTG	141
TGT-F	CACGACGTTGTAAAACGACGTGGTAATTGGAACCTGT	158
TTT-R	CTGCAGTTACTCACCAGAAA	221
TTC-R	CACGACGTTGTAAAACGACCCTGCAGTTACTCACCTCGAA	241
*Kdr*-F	TGCCACTCCGTGTGTTTAGA	325
*Kdr*-R	GAGCGATGATGATCCGAAAT	325
Super *Kdr*-F	GGCCGACCCTGAACTTACTC	450
Super *Kdr* R	GAAAATGCGGCCATCTTAGT	450

### Statistical Analysis

WHO (1998) criteria were used to evaluate the resistance or susceptibility of the tested mosquito populations (<80% mortality: resistance, 80–98% mortality: probable resistance; >98% mortality: susceptibility) [39]. Univariate analysis of variance (ANOVA) was conducted using arcsin transformation of mosquito mortality rate to determine among-population differences in mosquito mortality rate in the insecticide susceptibility bioassay. The *kdr* allele frequency was calculated in each population and statistical differences among populations were examined using the t-test. ANOVA also was used to examine whether monooxygenase and GST activity varied among populations.

Stepwise multivariate logistic regression and linear regression analyses were conducted to determine the role of *kdr* mutations and the level of detoxification enzymes on deltamethrin resistance. Stepwise regression is a procedure for automatically selecting independent variables in a stepwise manner to maximize the squared multiple correlations coefficient (*R* ^2^) (for linear regression) or the likelihood (for logistic regression) of the dependent variables against a set of independent variables. In the present study, the dependent variables were the mosquito knockdown time and survival status (dead or survived within 24-hr recovery period) for stepwise multiple linear regression or the stepwise logistic regression, respectively. In all tests, independent variables were the three *kdr* genotypes and the activity levels of monooxygenases and GSTs. A total of three *kdr* genotypes were considered: 1) homozygous wild type (TTG/TTG), 2) heterozygous mutations (TTG/TTT, TTG/TTC or TTT/TTC), and 3) homozygous mutations (TTT/TTT, TTC/TTC or TTT/TTC).

### Ethics Statement

No specific permits were required for the described field studies. For mosquito collection in rice paddies, oral consent was obtained from field owners in each location. These locations were not protected land, and the field studies did not involve endangered or protected species.

## Results

### Deltamethrin Susceptibility Bioassay

**Figure 2:**
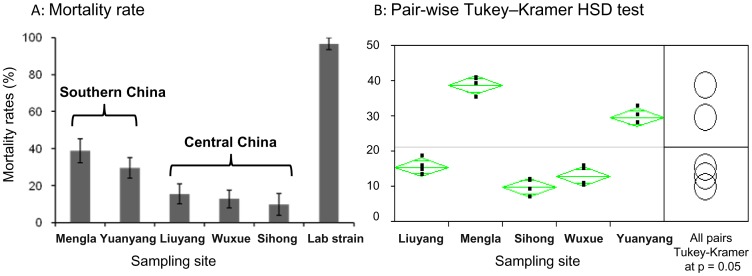
Mortality rates of *Anopheles sinensis* in standard WHO tube bioassays. A: after exposure to 0.05% deltamethrin test papers and 24 hr recovery period. B: pairwise Tukey–Kramer HSD test for statistical significance in mortality rates. The vertical bar stands for standard error of the mean.

All five tested populations were resistant to deltamethrin, with mortality rates ranging from 9.8% to 38.8% (Fig. 2A). ANOVA analysis found significant among-population variation in mortality rates (F_4, 15_ = 97.2, *P* < 0.0001). In particular, the two populations from Yunnan province in southern China (Mengla and Yuanyang, 29.6%–38.8%) had significantly higher mortality rates than populations from central China (Liuyang, Sihong, and Wuxue, 9.8%–15.0%) (Fig. 2B). The *An. sinensis* susceptible laboratory strain showed 100% mortality, confirming the quality of resistance test papers.

### *Kdr* Gene Sequencing and Allele-specific PCR (AS-PCR) for *kdr* Genotyping

**Figure 3:**
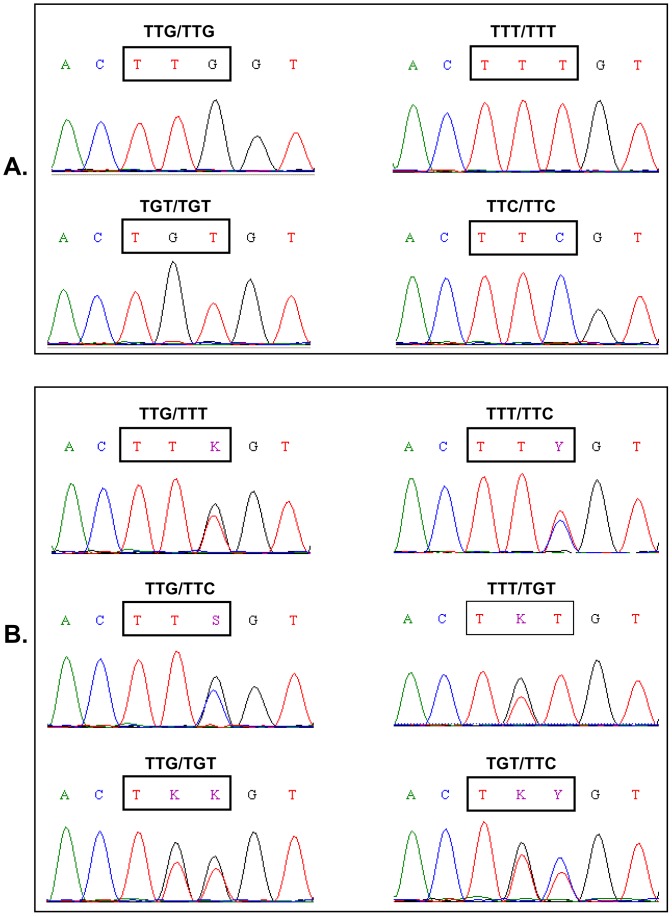
Examples of nucleotide sequence chromatograms of *kdr* genotypes detected in *Anopheles sinensis* from China. The position at codon 1014 of the *para*-type sodium channel gene is indicated by a rectangle box. **A:** four types of homozygote genotypes detected; and **B:** six types of heterozygote genotypes detected (K = G/T; Y = T/C; S = G/C).

**Figure 4:**
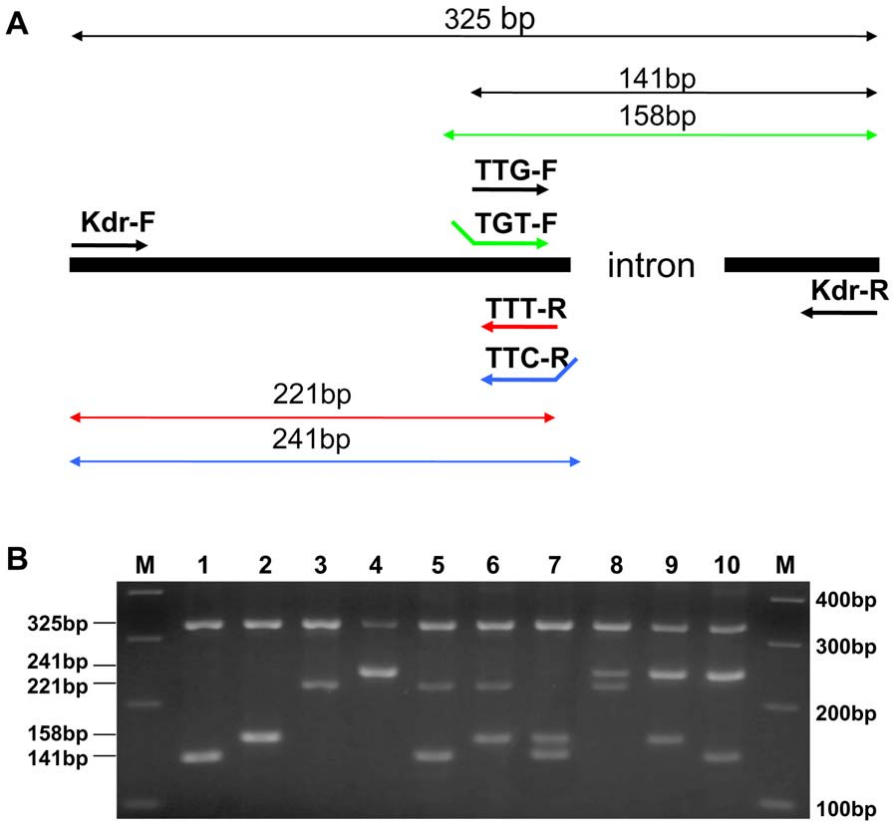
Allele-specific PCR (AS-PCR) for *kdr* genotyping in *Anopheles sinensis*. **A:** Schematic representation of the primer location and predicted size of the PCR product. Arrow indicated the location of PCR primers in which sequences are reported in Table 1. Primer pair TTG-F and *kdr*-R amplifies a 141 bp fragment for the wildtype allele (for codon TTG). Primer pair TGT-F/*kdr*-R yields a 158 bp fragment for L1014C allele (codon TTC). Similarly, primer pair *kdr*-F/TTT-R and *kdr*-F/TTC-R leads to amplification of a 221 bp and 241 bp fragment diagnostic to the L1014F allele (for codon TTT and TTC), respectively. **B:** An example of AS-PCR gel electrophoresis. Lane M: 100 bp DNA ladder; lanes 1–4: homozygote for the codons TTG, TGT, TTT, and TTC, respectively; lanes 5–10: six heterozygous individuals (TTG/TTT, TGT/TTT, TTG/TGT, TTT/TTC, TGT/TTC, and TTG/TTC).

A 325 bp fragment of the *para*-type sodium channel gene including position 1014 and a 450 bp fragment including position 918 were sequenced from 30 *An. sinensis* individuals in each of the five populations. The wild-type *kdr* codon sequence spanning position 1014 was TTG. Three types of *kdr* mutations were detected: two (TTT and TTC) leading to a change from Leucine to Phenylalanine and one (TGT) leading to a Leucine to Cysteine substitution. A total of ten genotypes were identified in the five populations (Fig. 3). No mutations spanning position 918 were detected. Based on these sequence data, an AS-PCR protocol for the identification of *kdr* mutations was developed (Fig. 4). The AS-PCR method showed 98.9% sensitivity and 87% specificity with respect to sequencing results. The AS-PCR method was used for *kdr* genotyping of 456 additional individuals.

### Distribution of *kdr* allele Frequencies in *An. sinensis* Populations

*Kdr* genotyping was performed on both susceptible (dead) and resistant (alive) mosquitoes recovered from the WHO bioassays for deltamethrin resistance. No *kdr* mutations were detected in the two populations from southern China (Mengla and Yuanyang) despite high levels of phenotypic resistance. An opposite situation was found in three populations from central China ((Liuyang, Wuxue, and Sihong), where deltamethrin-resistant mosquitoes carried a high frequency (88.5–94.8%) of both the L1014F and the L1014C mutations (Table 2). *Kdr* mutations were identified also in susceptible mosquitoes from populations in central China, but at a significantly lower frequency than the wild type allele (Paired t-test, t = 7.79, d.f. = 2, P<0.01).

**Table 2:** Frequencies (in percentage) of *kdr* alleles in relation to mosquito survival phenotype determined by the deltamethrin susceptibility bioassay in five *Anopheles sinensis* populations from China.

Population	Province	Survival status after24 hr recovery	Sample size (n)	L1014 (TTG)	L1014C (TGT)	L1014F (TTT+TTC)	Population *kdr* mutation frequency (TGT+TTC+TTT)
Mengla	Yunnan	Alive	57	100	0	0	0
		Dead	39	100	0	0	
Yuanyang	Yunnan	Alive	65	100	0	0	0
		Dead	25	100	0	0	
Liuyang	Hunan	Alive	121	9.5	12	78.5 (74.0+4.5)	88.5
		Dead	25	22	14	64.0 (62.0+2.0)	
Wuxue	Hubei	Alive	125	0.9	18.2	80.9 (72.7+8.2)	94.8
		Dead	22	15.9	31.8	52.3 (52.3+0.0)	
Sihong	Jiangsu	Alive	112	7.1	10.7	82.1 (75.0+7.1)	91.7
		Dead	15	16.7	23.3	60.0 (60.0+0.0)	

### Metabolic Enzyme Assay

Monooxygenase and GST activity varied both within and among the five populations tested (Table 3). The average GST activity was 0.23 umoles cDNB/min/mg protein in the laboratory strain and 0.39 umoles cDNB/min/mg protein for the five field populations. The GST activity was significantly higher in the three populations from central China (Liuyang, Wuxue and Sihong) than in the laboratory susceptible strain (F_4, 15_ = 4.04, P<0.05) (Table 3). The Mengla and Yuanyang population from southern China exhibited similar GST activity to the laboratory strain. The monooxygenase activity was two to three folds higher in the two populations from southern China than in the laboratory population (F_4_, _15_ = 25.2, P<0.0001). The three populations from central China showed comparable monooxygenase activity to the laboratory strain (Table 3).

**Table 3:** Glutathione s-transferases (GSTs) and monooxygenases activity in *Anopheles sinensis* female adults in five populations in China when compared with lab susceptible strain.

Population	Sample size (n)	GSTs (umoles) cDNB/min/mg protein	Monooxygenases (pmoles)7-HC/min/mosquito
**Southern China**			
Mengla	96	0.28 ± 0.07 a*	50.94 ± 2.80 a
Yuanyang	90	0.32 ± 0.08 a	80.41 ± 3.99 a
**Central China**			
Liuyang	146	0.41 ± 0.60 b	28.88 ± 2.87 bc
Wuxue	147	0.47 ± 0.08 b	27.51 ± 1.63 bc
Sihong	127	0.46 ± 0.09 a	28.58 ± 1.58 bc
Lab strain	100	0.23 ± 0.04 c	25.68 ± 0.75 c

* Each value represents the Mean ± Standard Error (SE). In every column that ends with the same letter, there is no statistical difference from comparison using the Duncan multiple range test at a confidence level of 95%.

### Role of *kdr* Genotypes and Metabolic Detoxification Enzyme in Resistance

**Figure 5:**
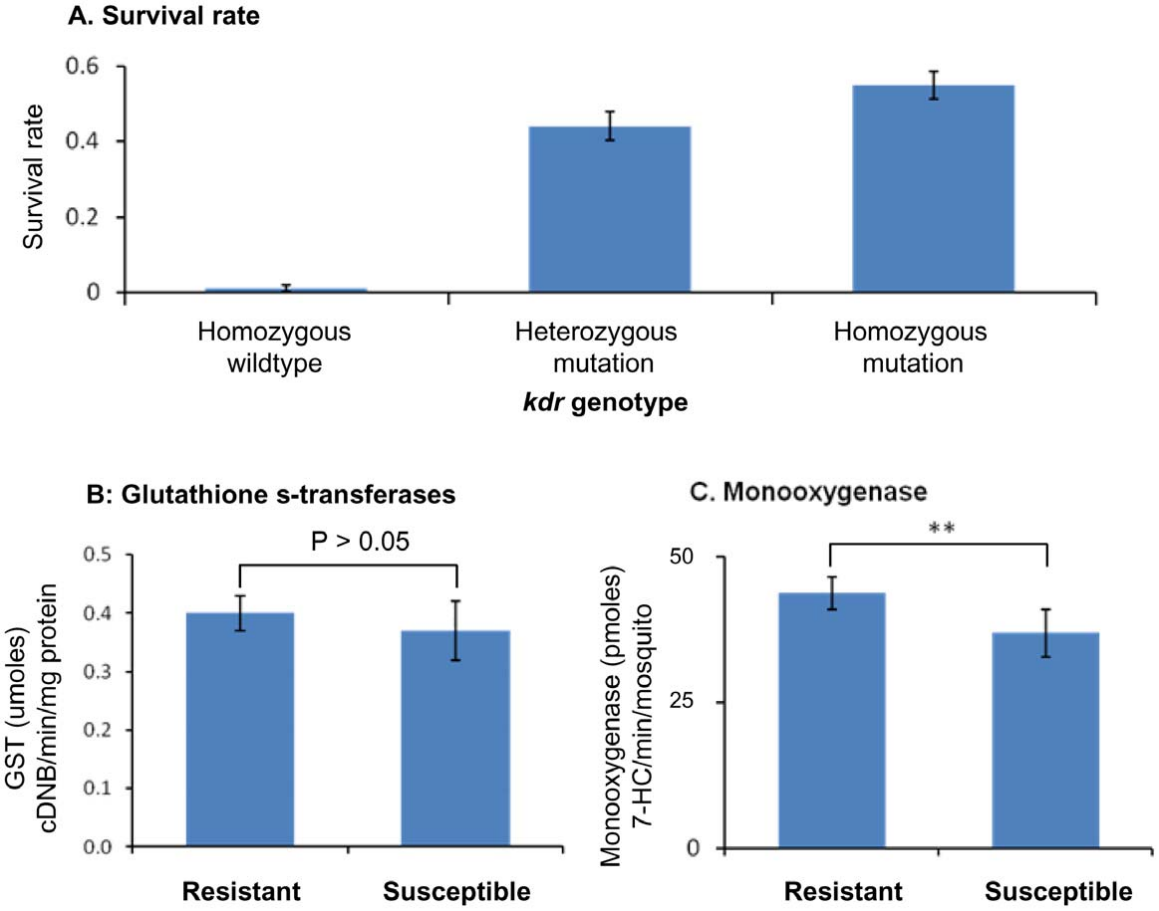
Relationship between *kdr*, metabolic detoxifying enzyme activity and pyrethroid resistance in *Anopheles sinensis.* Association between *kdr* genotypes and survival rate of mosquitoes after the standard WHO tube deltamethrin susceptibility bioassay (**A**), and variation of resistant (survived the bioassay) and susceptible (died in the bioassay) individuals in glutathione s-transferases activity (**B**) and monooxygenases activity (**C**). **, P < 0.01.

We conducted three analyses to determine the relative contribution of the different *kdr* genotypes and the activity of monooxygenase and GSTs enzymes on deltamethrin resistance. First, we tested whether the frequency of the different *kdr* genotypes varied between phenotypically -resistant and -susceptible mosquitoes in the three populations from central China (Liuyang, Wuxue and Sihong). The percentage of resistant mosquitoes that were homozygotes for the wild-type *kdr* genotype was low (1.1%). Total of 44.0% and 54.8% resistant mosquitoes showed *kdr* genotypes with mutations in a heterozygote or homozygote state, respectively (Fig. 5A). Second, we determined whether the activity of monooxygenases and GSTs were different between phenotypically - resistant and -susceptible mosquitoes. GST activity was not significantly different between resistant and susceptible individuals (Fig. 5B) in all tested populations. Monooxygenase activity was significantly higher in resistant than in susceptible mosquitoes (t-test, P<0.05) (Fig. 5C) in all tested populations.

Finally, we performed a stepwise multivariate logistic regression and a linear regression analysis which considers *kdr* genotypes, GSTs and monooxygenase activities simultaneously to minimize the effects of collinearity between the two variables. Stepwise linear regression analyses with knockdown time as the dependent variable identified *kdr* mutations in either a homozygous or heterozygous state and levels of monooxygenase activity as the significant independent variables (ANOVA *F*_3, 293_ = 13.078, *P*<0.0001) (Table 4). In our model, positive standard coefficients were identified for homozygous *kdr* mutations and monooxygenase activity, with the latter showing higher standardized coefficient. This indicates a positive effect on resistance, with monooxygenase activity having a stronger impact (Table 4). Consistent with the knockdown time analysis, stepwise logistic regression analysis using survival status as the dependent variable selected homozygous mutations and monooxygenase activity as the significant variables. High odds ratio for monooxygenase activity suggests that mosquitoes with high monooxygenase activity were far more likely to be deltamethrin resistant (Table 5).

**Table 4:** Summary results of stepwise multivariate regression analysis on knockdown time in *Anopheles sinensis*.

Effect	Coefficient	Standard Error	Standard Coefficient	*t* value	P
Constant	2.628	0.072	0	36.314	<0.001
Heterozygous mutation	−0.207	0.083	−0.152	−2.478	0.014
Homozygous mutation	0.155	0.054	0.174	2.853	0.005
Monooxygenase	2.323	0.638	0.2	3.644	<0.001

Notes: Only significant variables are presented.

**Table 5:** Stepwise logistic regression for factors significantly associated with deltamethrin resistance in *Anopheles sinensis.*

Effect	Coefficient	Standard error	Odds Ratio (95% CI)	*t* value	P
Constant	−0.596	0.393		−1.515	0.1300
Homozygous mutation	1.136	0.292	3.11 (1.75, 5.52)	3.887	< 0.0001
Monooxygenase	14.366	3.951	19.67 (2.50, 189.42)	3.636	< 0.0001

Notes: Only significant variables are presented.

## Discussion

This study examined phenotypic resistance to deltamethrin in *An. sinensis* from China in relation to mutations in the insecticide target site and levels of detoxification enzymes. All five studied populations were highly resistant to deltamethrin, with particularly high levels (>85%) in populations from central China. When target site mutations and levels of detoxification enzymes were tested in phenotypically- resistant and -susceptible mosquitoes, major differences were found between the populations from southern and central China. Interestingly, no *kdr* mutations were found in the two populations from southern China, but high mutation frequencies (88.5–94.8%) were identified in the three populations from central China. In these populations three *kdr* genotypes were identified, leading to either a L1014F or a L1014C substitution. We did not detect any L1014S mutation as previously reported in *An. sinensis* populations from the Republic of Korea and Vietnam [17], [31], [47], [48]. Regarding metabolic detoxification mechanisms of resistance, GST activity was not detected as a significant variable associated with deltamethrin resistance in the multiple regression analyses. On the contrary, monooxygenase enzymes were significantly correlated to phenotypic resistance in all tested populations, with particularly high levels of enzyme activity in populations from southern China. The results show that metabolic detoxification mechanisms are wide-spread in *An. sinensis* populations from southern and central China, but target site mutations confined to central China suggest that different resistance mechanisms can arise in locally different settings, probably in relation to different selection pressures. Additionally, we noticed higher levels of phenotypic resistance in populations from central China, where both *kdr* and detoxification mechanisms were identified, suggesting an additive effect of target site mutation and metabolic detoxification on phenotypic resistance.

Yunnan is a typical longitudinal range-gorge region with complex terrain and climate, where great mountains and deep valleys run parallel and connect the Indochinese peninsula and the inner area of western China [49]. The mountain ranges in the longitudinal range-gorge region of Yunnan effectively restrict gene flow in the latitudinal direction. Population genetic studies of *An. sinensis* revealed the role of the geographical barriers in obstructing gene flow among the mosquito populations [2], [5]. In the mountainous regions of southern China, agricultural crops are diverse and insecticide use has, traditionally, not been intensive. On the other hand, insecticide use has been intense in central China, with several rounds of spray *per* year [16]. Rice is the most widely planted crop in this region and rice paddies are the major breeding sites of *An. sinensis* mosquitoes. It will be interesting to test for traces of insecticides in breeding site water as it has been suggested that phenotypic resistance in adults is due to biological influence of the breeding sites on mosquito immature stages [50].

All specimens used in the bioassay were adult mosquitoes reared from larvae collected from the field. Although this was very time consuming and labor intensive, this design allowed using mosquitoes of fixed age (3 days post emergence) and with known physiological status (no blood feeding) for the bioassay, thus minimizing the confounding effects of age or blood-feeding status on resistance phenotype and subsequent metabolic enzyme activity measurement. Because the resistance bioassays were conducted with individual mosquitoes, we could measure the precise knockdown time and resistance status within the 24-hr recovery period. This enabled us to accurately determine the relationship between knockdown time, resistance status, *kdr* genotypes and monooxygenase and GST activity. Our results indicate that overall knockdown time in bioassays is a good approximation of pyrethroid susceptibility [51], [52]. None of the resistant mosquitoes were knocked down during the 60 min exposure time. This suggests that a complete loss of knockdown is indicative of deltamethrin resistance. On the other hand, susceptible mosquitoes were being knocked-down and showed high levels of variation in the knockdown time. The majority of susceptible mosquitoes were dead within 10 hours after the end of the bioassay. This result is possibly related to the slow killing effect of deltamethrin [53] and suggests phenotypic plasticity, which should be further investigated.

Considering the high pyrethroid resistance in *An. sinensis* populations in China, resistance management becomes a particularly important issue. Insecticide rotation or synergistic uses of different classes of insecticides to enhance the killing effects are being considered as alternative methods of vector control to reduce the human-vector contact rate and to slow down the spread of resistance genes [54]–[57]. Therefore, resistance to multiple classes of insecticides and the role of selection by insecticides used for both agricultural pest control and public health pest control are also important topics for future research. Such knowledge will help guide rational use of insecticides in both public health and agriculture. Additionally, the identification of widespread metabolic detoxification mechanisms with the confinement of *kdr* mutations to central China suggests that the development of reliable resistance surveillance tools should focus on monooxygenase metabolic detoxification mechanisms.
